# Brief report: caregivers’ well-being in families with neurodevelopmental disorders members during COVID-19: implications for family therapy

**DOI:** 10.3389/fpsyt.2024.1409294

**Published:** 2024-08-02

**Authors:** Daniela Sousa, Ana Ferreira, Joana Sequeira, Marilyn J. Monteiro, Marco Simões, Miguel Castelo-Branco

**Affiliations:** ^1^ Coimbra Institute for Biomedical Imaging and Translational Research (CIBIT), Institute for Nuclear Sciences Applied to Health (ICNAS), University of Coimbra, Coimbra, Portugal; ^2^ Faculty of Medicine, University of Coimbra, Coimbra, Portugal; ^3^ Instituto Superior Miguel Torga (ISMT), Coimbra, Portugal; ^4^ PhD Licensed Psychologist, St. Petersburg, FL, United States; ^5^ Centre for Informatics and Systems (CISUC), University of Coimbra, Coimbra, Portugal

**Keywords:** COVID-19, autism, neurodevelopmental disorders, caregiver’s well-being, family therapy

## Abstract

Neurodevelopmental disorders affect the lifespan of diagnosed individuals and their families. COVID-19 challenged these families with daily routine unpredictability requiring rapid adaptations. Moreover, associations and schools were closed, leaving these families without regular social support. Here, we investigate which individual and family factors can predict the caregiver’s depressive state and overall burden. An online study took place between 2021 and 2022. A total of 32 caregivers (30 women; 48 ± 8.22 years old; range 26 to 63 years old) reported having a family member with a neurodevelopmental disorder, the majority diagnosed with autism spectrum disorder. Caregivers responded to a protocol to assess the burden, resilience, depressive, anxious, and stress symptomatology, as well as the behavior of the diagnosed individual. Hierarchical multiple regressions were performed to identify protective and risk factors for the caregivers’ well-being. Caregivers’ depressive state was explained by 29.3% of the variance of the family cohesion factor, indicating that high levels of balanced family cohesion represent a crucial protective factor for reducing the caregiver’s depressive state. Additionally, overall caregiver burden was explained by 17.8% of the variance due to self-perception and 26.4% due to family cohesion, with the caregiver’s self-perception playing an important protective role in the overall perception of burden. The proportion of male and female respondents seems to corroborate the significant role of women in caregiving. These results emphasize the importance of considering both individual and family factors of caregivers during interventions, which have implications for family therapy with families of members diagnosed with neurodevelopmental disorders, specifically with autism.

## 1 Introduction

Neurodevelopmental disorders (NDDs) are manifested typically early in life during the development. The American Psychiatric Association in the Diagnostic and Statistical Manual of Mental Disorders (5^th^ edition, DSM-5) proposed a broadening of categories to cover NDDs, including the intellectual, communication, autism spectrum, attention-deficit/hyperactivity, specific learning, motor, tic, and other NDDs without specifications. These disorders are characterized by impairments in personal, social, academic, or occupational functioning (1). In addition, they are lifelong disorders and require an informal care provision by family members that are many times invisible to others. Therefore, parents navigate between health services, multiple therapies, educational responsibilities, and emotional and behavioral problems single-handedly (2).

Studies showed that having a family member with an NDD was associated with negative outcomes for caregivers, which include burnout, emotional exhaustion, depression, and physical fatigue (3). Usually, these parents experience high levels of stress compared with other chronic diseases (2, 4, 5). Additionally, it is known that stress is associated with parenting self-efficacy perception and influences the intervention outcomes of the individual with a NDD diagnosis (6). However, different family members, such as siblings, can assume caregiver roles. Sibling-focused parentification, where siblings support a brother/sister with an NDD emotionally or instrumentally, can lead to both positive (e.g., responsibility, high self-efficacy, empathy) and negative outcomes (e.g., rejection, guilt, anxiety, and depression) (7–9). The father’s role appears to be less involved (10), with a lower propensity for depressive symptoms (7) and a higher perception of competency (11). Their distress may manifest differently and warrants further exploration (12). In most studies, the focus on caregivers shows that women are more represented (5, 7–9). The COVID-19 quarantine challenged these families with an array of problems, potentially further increasing caregivers’ stress levels (2, 13). People with NDD find stability in routines by knowing what is expected at each moment (5). The unpredictability due to the COVID-19 context led to anxiety, frustration, and emotional breakdowns (2, 14, 15). Caregivers had to deal with an increased incidence of these states and the unexpected changes associated with the closure of schools and other support services, which before allowed them to have some respite care from informal care provision. Furthermore, the circumstances of COVID-19 potentially induced an environment where the boundaries between the caregiver’s roles (e.g., professional, parental) were diluted, which may contribute to an increase in feelings of burden.

Home quarantine and isolation challenged families dealing with worsening symptoms (5, 16), behavioral and emotional regulation, and routine adherence difficulties (2) with interruptions of support services (5). The exposure to COVID-19 may have contributed to intensified stress responses leading to aggression and irritability (2), and, in autism, with a higher probability of using maladaptive coping behaviors, which exacerbates the characteristic autistic behaviors (6, 17). A low level of support and sharing in coparenting is a risk factor for parental distress (18). Adults with NDD have reported higher rates of anxiety symptoms but may nevertheless show improvements in depression levels (due to less exposure to negative feedback; relief of day-to-day demands) (19). NDDs may unveil maladaptive feedback loops that can occur during challenging times within a familiar context of informal care provision. Moreover, a study found a relationship between the worsening of the symptomatic changes in children and higher parental distress levels, which may lead to an escalating positive feedback loop (5), and this finding seems to be independent from the type of NDD diagnosis (18). Therefore, the families of people with NDDs are at greater risk of being negatively impacted by the pandemic, as an external source of family stress (15) describing, e.g., feelings of loneliness (18). However, the more time with the family and strengthening relationships may on the other hand have helped to get to know better their child and the experience of closeness were positive aspects reported (18).

Families faced the early challenging time of COVID-19 with preoccupations about future lockdowns, stability of living situation, and scarce financial resources for day-to-day expenses (5). People with NDDs struggled to comply with COVID-19 recommendations (e.g., social distancing; use of masks) (5). Studies found that the interruption of routines and the high stress related to the lockdowns and restrictions were associated with a higher impact on child and parent well-being (5). Another study showed that during COVID-19 when the mother was suffering from a depressive state, a negative interaction with routines and children’s maladaptive behaviors were identified (14). This type of interaction was seen previously in other natural disasters and even worse during emergencies (14).

It is acknowledged that individual characteristics, such as child behavior problems, maternal stress, coping style, and familiar functioning dimensions, namely, family cohesion, have been consistently related with depressive symptoms on caregivers (20). Therefore, the caregiver’s depressive state and the overall relational burden should be understood in the context of the informal care provision relationship, which is markedly demanding in a highly stressful context, such as the COVID-19 pandemic. Therefore, based on previous evidence, we expected to identify whether individual factors, such as self-perception, and family factors, such as family cohesion, could have either a protective or risky impact on the development of caregivers’ depressive states and perception of overall burden. Additionally, we anticipated identifying a gender tendency associated with the caregiving role, with a greater representation of women.

## 2 Methods

### 2.1 Procedure

The study was approved by the Faculty of Medicine ethics committee from the University of Coimbra (Portugal). Written informed consent was obtained from the respondents. The online study took place from April 2021 until March 2022 on the Neurohab platform (21). The sociodemographic questionnaire was developed to explore the COVID-19 pandemic impact on individual and family daily life (Sociodemographic questionnaire in **Supplementary Materials**). Additionally, the protocol included a variety of self-report instruments to evaluate the caregiver’s psychopathology, resilience dimensions, burden, and maladaptive behavior of the individual with a NDD. The overall online study took approximately 45 min to fill out.

### 2.2 Participants

There were 32 respondents (30 women and 2 men; mean (*M*) age = 48 years old, standard deviation (*SD*) = 8.22) reported having a family member with a prior formal NDD diagnosis, according to the DSM-5 classification. However, only 27 caregivers completed the entire protocol. This sample originates from 702 adults who responded to a larger online protocol about COVID-19 pandemic. The degree of relatedness of caregivers to the member diagnosed with an NDD, as well as the percentages of NDD categories, is presented in **Table 1**. Down syndrome was reported and included, as it is considered a neurodevelopmental disorder due to the presence of neurodevelopmental abnormalities (22). It is the most frequent cause of intellectual disability and has a high co-occurrence with autism spectrum disorder (23–25). Autism spectrum disorder was the diagnosis most frequently reported in our sample. The age of the family member with the NDD was on average 22.78 years old (*SD* = 19.80; range 4 to 86 years old). Sample characterization details can be found in **Supplementary materials**.

**Table 1:** Degree of relatedness between the caregiver and the member with a neurodevelopmental disorder and the neurodevelopmental disorders diagnoses.

Degree of relatedness	Father or mother	Sister or brother	Son	Stepmother	Other (missing response)	
***n*** **(Percentage %)**	23 (71.9%)	1 (3.1%)	6 (18.8%)	1 (3.1%)	1 (3.1%)	
**Diagnoses (DSM-5)**	**Autism spectrum**	**Hyperactivity and/or attention deficit**	**Ticks disorder**	**Down syndrome**	**Intellectual disability**	**Motor disorder**
***n*** **(Percentage %)**	19 (59.4%)	2 (6.3%)	1 (3.1%)	6 (18.8%)	2 (6.3%)	2 (6.3%)

### 2.3 Materials

Sociodemographic questionnaire and protocol measures are presented and additional information can be found in **Supplementary Material, Table S3**.

#### 2.3.1 Depression Anxiety Stress Scales 21 items

Depression Anxiety Stress Scales (26, 27) (DASS-21) were developed to measure anxiety, depression, and stress. Depression was defined as a result of motivation and self-esteem loss, anxiety as a long-lasting state of anxiety and intense responses to fear, and stress as excitation states and persistent tension (e.g., difficulty relaxing, irritability, and agitation) combined with frustration and disappointment. These scales assumed that mental disorders are dimensional, and the overall scale is composed by 21 items about negative emotional symptoms. The caregivers’ rated the affirmations by evaluating on a four-point Likert scale of severity/frequency related to if they have experienced that symptom during the past week. These scales provide a score per scale and a total score, with higher scores reflecting higher levels of negative affective state.

#### 2.3.2 Resilience Scale for Adults

The Resilience Scale for Adults (28–30) (RSA) assessed resilience as a multidimensional concept theoretically based on the assumptions of protective resources in three areas: individual psychological abilities; family context and support; and external support systems that provide an efficient coping and adjustment (11). Therefore, RSA is composed of 33 items distributed across different dimensions, namely, self-perception defined as the self confidence about abilities, judgments, personal agency, and real expectations; future planning which is the ability to plan, with an optimistic vision, and is oriented by clear and realistic goals; social competencies related to the flexibility within social relationships, as friendships, being at ease in social contexts and the positive use of humor; structured style which is about the ability to have a routine, good time management, and preference for goals and plans; family cohesion which is associated with shared values, appreciation in sharing time, loyalty, optimistic vision of the future, and a feeling of mutual appreciation and support; social resources which are related to social support when individuals have a trusted person outside the family to whom they can ask for assistance. Caregivers must choose within 1 to 7 the response that suits them. This measure has a score per dimension and a total score, with higher results indicating better resilience.

#### 2.3.3 Revised Burden Measure

Revised Burden Measure (31, 32) (RBM) is used to evaluate the caregiving burden and gratifications related to the informal care provided to individuals with chronic health problems. This scale is based on a relational and social context and includes the evaluation of positive affect related to the care provision, which can coexist with negative affect. The questionnaire has 22 items divided in four subscales: the relationship burden which is defined as the caregiver’s perception of demands required by the person, which are not aligned with the medical condition; the objective burden as a negative state which results from the care provided during the caregiver’s free time; the subjective burden as the global negative affect associated with the care provision; and the gratification defined as the positive psychological state associated with informal care. Caregivers are invited to reflect on how their lives changed as a result of care provision on a 5-Likert scale. This measure gives a score per subscale and a burden overall score. The higher scores in the burden and/or uplifts scales indicate a significant modification in that caregivers’ life dimensions.

#### 2.3.4 Aberrant Behavior Checklist-Community

The Aberrant Behavior Checklist – Community (33, 34) (ABC-C) evaluates the presence and variety of maladaptive behaviors across five subscales: Irritability, Agitation, Crying; Lethargy, Social Withdrawal; Stereotypic Behavior; Hyperactivity, Noncompliance; and Inappropriate Speech. The 58 items are rated on a 4-point Likert scale, with higher scores indicating more severe problems (34). The rater is asked to consider behaviors that have interfered with the individual development, functioning, and/or social as problematic during the last 4 weeks (35). The scores are obtained per subscale (36).

### 2.4 Data analysis

All statistical analyses were performed in IBM Statistical Package for the Social Sciences (SPSS), Version 25. Descriptive analyses were performed to explore the prevalence of caregivers’ symptomatology, burden, and resilience dimensions and behavior dimensions of the individual with an NDD. The normality assumption for the instrument’s dimensions selected was verified using the Shapiro–Wilk test. The variables depression (DASS-21), the burden (RBM), and the irritability (ABC) were not normally distributed (p <.05). Two exploratory analysis using hierarchical multiple regressions were performed to identify protective and risk factors for the caregivers’ well-being (**Supplementary Material**, Exploratory Analysis). Hence, two final multiple regressions were performed, building upon prior exploratory analysis investigating statistical significance of addition of predictors identified in the previously explored models (**Supplementary Material**, Hierarchical Multiple Regression Assumptions). Additionally, a binomial test was done to compare if there is a difference in proportions between groups regarding caregivers’ gender.

## 3 Results

### 3.1 Individual and family factors characterization

**Figure 1:**
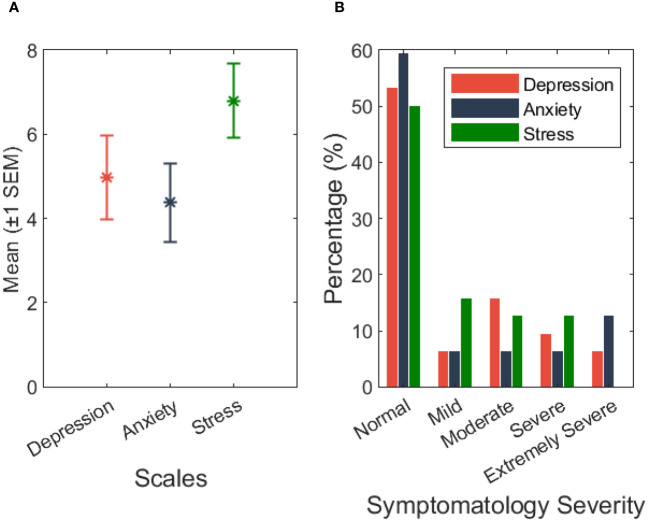
**(A)** DASS-21 mean (*M*) ± 1 standard error of mean (*SEM*) by classification (please see **Table S3, Supplementary Material**). **(B)** DASS-21 characterization of symptomatology severity (*N* = 29) in percentage (%).

The results obtained with the DASS-21 revealed that 12 (37.6%) caregivers reported a depressive state ranging from mild to extremely severe. Concerning anxiety, 10 (31.4%) caregivers reported levels between mild to extremely severe, and in the stress scale, 13 (40.6%) caregivers reported mild to severe levels of stress. **Figure 1** details the symptomatology severity across DASS-21 scales.

**Figure 2:**
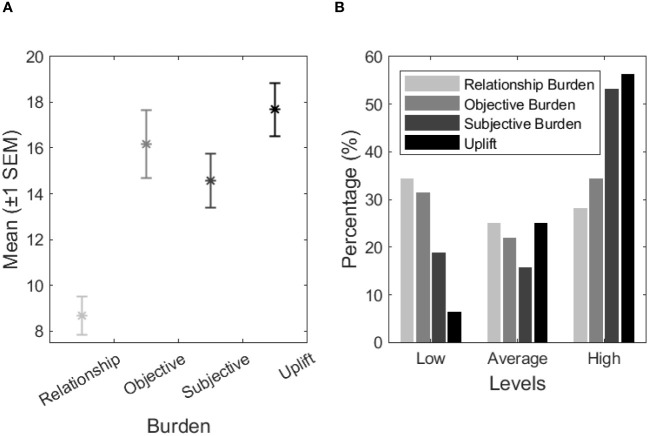
**(A)** RBM *M ± SEM* categorization classification (please see **Table S3, Supplementary Material**). **(B)** RBM results characterization on levels of burden and uplift or gratification by percentage (%) (*N* = 28).

The RBM results showed that 17 (53.1%) caregivers felt an average to high level of relationship burden and 18 (56.3%) an average to high level of objective burden. In the subjective burden, many caregivers felt an average to high level of burden (*n* = 22, 68.7%), and most of them felt an average to high gratification associated with the informal care provision (*n* = 26, 81.3%). **Figure 2** synthetizes the results obtain in the RBM.

Caregiver’s resilience dimensions and behavioral characterization of the individual with NDD can be found in **Supplementary Material** (**Supplementary Figures S1**, **S2**).

### 3.2 Caregiver’s depressive state and overall burden prediction

The first model included the family cohesion as a predictor of caregiver’s depressive state, *F (*1, 25) = 10.351, *p* <.05. We found that the family cohesion led to a *R^2^* of .293. Therefore, 29.3% of the variance explained in the depression scores can be attributed to the family cohesion. This result indicates that family cohesion was significantly negatively associated with the caregiver’s depressive sate (*B* = −3.413, *t* = −3.217, *p* = .004). Therefore, the change in one unit for family cohesion score will result in –3.413 (95% confidence interval CI, −5.598 to −1.228) in the value of depression scores. The addition to the model of subjective burden led to a statistically significant model, *F (*2, 24) = 6.503, *p* <.05. However, the addition of this predictor did not prove to be significant.

Regarding the caregiver’s overall burden prediction, we introduce in the first model the self-perception, *F (*1, 25) = 5.429, *p* <.05. This individual factor explains 17.8% of the overall burden. The family cohesion addition to the second model led to a statistically significant increase in *R^2^* of .264, *F (1*, 24) = 11.366, *p* <.05, and maintaining this second model statistically significant, *F (2*, 24) = 9.523, *p* <.001. These results show that caregiver’s overall burden prediction is intimately and negatively associated with self-perception (*B* = −6.233, *t* = −2.330, *p* = .028) and family cohesion (*B* = −11.547, *t* = −3.731, *p* = .003). Hence, the change in one unit for self-perception and family cohesion will result in –6.233 (95% CI, −11.742 to −.724) and –11.547 (95% CI, −18.615 to −4.478) in the value of overall burden, respectively. **Table 2** summarizes these results.

**Table 2:** Hierarchical Multiple Regression results for caregiver’s depressive state on the left side of the table and Hierarchical Multiple Regression results for caregiver’s overall burden on the right side.

	Depressive state				Overall burden				
	Model 1		Model 2			Model 1		Model 2	
Variable	*B*	β	*B*	β	Variable	*B*	β	*B*	β
**Constant**	23.531		12.748		**Constant**	68.200		108.470	
**FC**	−3.413	−.541	−2.148	−.341	**SP**	−6.233	−.422	−1.482	−.100
**SB**			.272	.314	**FC**			−11.547	−.606
***R^2^***	.293*		.351		***R^2^***	.178*		.442*	
***F***	10.351		6.503		***F***	5.429		9.523	
***ΔR^2^***	.293		.059		***ΔR^2^***	.178		.264*	
***ΔF***	10.351		2.170		***ΔF***	5.429		11.366	

*N* = 27, *p <.05, **p <.001.

FC, Family Cohesion; SB, Subjective Burden; SP, Self Perception.

### 3.3 Caregiver roles by gender

In this sample, we have a statistically significant difference in caregivers’ gender with a .94 proportion of women to a .06 proportion of men (*p* <.001).

## 4 Discussion

The protective factors that can be resources against the development of psychiatric disorders (e.g., depression) have been seen as a significant force behind healthy adjustment to life stressors (e.g., financial issues, maladaptive behaviors) or to regain and maintain mental health (11). It is known that families of children with NDDs, namely, the parents, have a high risk of presenting high levels of depression and anxiety, due to several stressors (e.g., diagnosis severity, relation with the child) (37). In this study, we investigated how individual and family factors can predict the levels of depression and the overall burden of caregivers to better understand which factors should be addressed to prevent and intervene with these families.

Our sample is aligned with the literature where women, specifically mothers, are usually the primary caregiver when special care is required in chronic diseases (16, 20, 32, 37). Previously to COVID-19, a meta-analysis identified that the relationship mother–child was a risk factor, and it was associated with higher rates of depression in mothers compared with fathers (37). Also, high ratings of depression were associated with low family support/cohesion, low maternal health, high levels of stress, less effective coping styles, and the presence of more than one child with NDD (20). These results highlight the importance of understanding the challenges of these families in “typical” periods versus “atypical” periods for family life, such as the COVID-19 pandemic.

Empirical evidence showed that there is a bidirectional and reciprocal effect between the challenges of caring a children or adult with NDD and the quality of the relationship, which reinforces the importance of family involvement (7–9, 16, 18). For example, in a systematic review and meta-analysis, the authors found that parents of children with autism had moderate effect sizes for elevated depression (37). In our study, we found that balanced family cohesion (which means a family cohesion that allows the individual and family development, reflecting the emotional bonding that family members have toward one another) to the prediction model of the caregiver’s depressive state increased the variance explained by this protective factor by 29.3%. This result emphasizes the importance of working with families in which there are family members with NDD. This finding was supported by the result obtained with the addition of family cohesion to the prediction model of the caregiver’s overall relational burden explaining 26.4% of the variance. Furthermore, balanced family cohesion can be a protective factor for the caregiver’s mental health and in the feeling of subjective burden resulting from the informal care provision. The family cohesion as a protective factor, which can alleviate the negative impact of life stress events, was previously reported (38). Hence, the alteration of burden perception may be a potential factor to improve caregiver’s adaptation to outcomes (31). The combination of individual and social factors, namely, resilience, which includes the family support or social competence, seems to be crucial to withstand life stress (11). Studies have shown that resilient individuals seem to cope more functionally and flexibly with stress, and these attributes are developed early in life, for example through secure attachment, and resilient women seem to elicit and provide more social support (11). Furthermore, family resilience includes family cohesion, which is a fundamental process associated with well-being during serious crises (38, 39). In addition, a literature review showed that during COVID-19, it was important to maintain a good and healthy communication and to find positive activities to do, between family members, to create a sense of togetherness, trust, cohesion, and happiness (39). Moreover, results in family cohesion during COVID-19 in families without family members with NDD showed evidence of increasing family cohesion strongly associated with health status, namely, families with highly balanced family cohesion promoting healthy behaviors (40). Contrarily, our results indicate that when there is an increase in the caregivers’ overall burden and depressive state, there is an association with low balanced family cohesion. Therefore, we hypothesized that during the COVID-19 pandemic, there was a rigidification of roles in those families, amplifying the perception of disconnection and burden without social and family support. Then, providing family support is specifically important since balanced family cohesion is perceived as a high sense of connectedness, affection, and support that allows positive individual growth with autonomy and effective development of family functions (40).

Lastly, it is important to underline those parents of individuals with autism brain style which are known to show more distress when compared with other NDDs (5). Additionally, the few studies that have explored the effect of COVID-19 pandemic on the well-being of parents of children with NDD highlighted the need to support these parents (5, 13, 18). Therefore, the creation of support services for caregivers’ mental health is crucial to avoid decreasing the quality of life and well-being (14). It is universally recognized that families are the constant in the individual’s life and are best suited to determine their family member’s needs. However, in challenging times, the coping skills of these families could suffer from the interaction effects of individual characteristics and family functioning. Therefore, it is of utmost importance to understand the individual and family factors that could be addressed during interventions with these families. Family therapy approaches are appropriate to respond to individual and family needs by creating a secure context for all family members to share their perspectives about problems. Narrative family therapy can be useful by creating narrative transformations that shape individual and family discourses. This approach proved to be effective in a wide range of mental health conditions (41). According to Monteiro (2021), this approach to support families in which a member has NDD, namely, autism brain style differences, has been less explored or developed. However, it may be appropriate for a range of NDDs considering the family life cycle stage. This approach allows the therapist to create the context to develop therapeutic conversations with the family about the strengths and differences of the individual with NDD based on descriptive strength language (42). Concurrently, in this process of shifting the narratives, the therapist will work on the caregiver’s self-perception. This resilience dimension includes self-confidence about abilities, judgments, personal agency, and realistic expectations. However, caregivers oftentimes have doubts about their capacities to respond adequately to the needs of their family member. Hence, caregivers can develop a negative self-perception which impact their sense of competency to deal with daily life challenges and this negative sense can be increased by external sources of stress. This process can create a powerlessness narrative which needs to be transformed into a narrative of competence during the therapeutic process. Therefore, the therapeutic conversations will focus on creating a plot of new emerging narratives about the past and present singularities, which will allow to create a sense of hope and competency for the future in the relational context. Additionally, it is in this process that a shift can occur in burden perception. Accordingly, by the therapist exploration of singularities, which refers to unique moments or exceptions that are in line with the family’s identity, we will promote novelty in individuals’ and families’ responses that will be amplified during the therapy (43). For these therapeutic conversations, it is important for the therapist to be aware of the narrative change dimensions which should be questioned and perturbed to open space for the emergence of these new stories (43). The construction of new storylines about the individual and the family, concerning the difficulties, feelings of appreciation, and support within the family context, can emerge. Additionally, an optimistic vision of the future can be co-constructed by having therapeutic conversations about the strengths of the individual with NDD and how the challenges can be overtaken by them, together. During this process, family and individual values are shared and amplified in session creating a sense of loyalty between family members, since all are aligned in a core dimension of family functioning, which means that family cohesion works along with therapy.

### 4.1 Limitations

This study has some limitations; that is, it is a cross-sectional study with a relatively low sample size, although one must consider that the base sampling is from a larger study. Additionally, there is a heterogeneous number of families regarding the NDD diagnosis according to DSM-5 classification. Moreover, we only have the response from the point of view of one caregiver, in particular given the larger adherence of women, and we used self-report measures, which reflect the subjective perception of the respondent. Moreover, we acknowledge the possibility of misunderstandings during the protocol completion. Therefore, these results should be interpreted with caution. Longitudinal online studies should be conducted to reach various family members, aiming to better understand the caregiving systems in NDDs in different times during normative crises (e.g., transition from adolescence to young adult) or unexpected (e.g., pandemic). Future research should include efficacy studies to provide evidence of the effectiveness of narrative family therapy in NDDs and to deepen our understanding of the long-term outcomes on family cohesion and the mental health of family members.

### 4.2 Future directions

Finally, our results point out at the individual level the importance of the caregiver’s self-perception, such as self-confidence to face daily life challenges, which can impact on burden perception. Additionally, on the family level, the sense of togetherness seems to be crucial for these families. These results support the importance of the relational context during an experience of high external stress, such as the COVID-19 pandemic. Future works and clinical practice should pay particular attention to the individual and family factors of caregivers of individuals with NDD, which can impact positively or negatively on their mental health.

## Data availability statement

The raw data supporting the conclusions of this article will be made available by the authors, without undue reservation.

## Ethics statement

The studies involving humans were approved by Comissão de Ética da Faculdade de Medicina, Universidade de Coimbra. The studies were conducted in accordance with the local legislation and institutional requirements. The participants provided their written informed consent to participate in this study.

## Author contributions

DS: Writing – original draft, Visualization, Writing – review & editing, Investigation, Conceptualization. AF: Validation, Writing – review & editing, Investigation. JS: Supervision, Writing – review & editing, Validation, Investigation. MM: Writing – review & editing, Validation, Supervision, Investigation. MS: Software, Writing – review & editing, Validation, Investigation. MC-B: Supervision, Project administration, Funding acquisition, Conceptualization, Writing – review & editing, Validation, Investigation.
