# Supplementary material for: Brief report: caregivers’ well-being in families with neurodevelopmental disorders members during COVID-19: implications for family therapy
Source: Front Psychiatry. 2024 Aug 2;15:1409294. doi: 10.3389/fpsyt.2024.1409294 (PMC11327121; doi:10.3389/fpsyt.2024.1409294)
Supplement: Supplementary file 1 [file DataSheet_1.pdf]

Supplementary Material

Sample Characterization

Regarding the civil status, our sample has 22 married respondents, 4 divorced, and 6 non-marital partnerships. Table S1 reports the sample educational level and professional situation. Out of the 8 unemployed respondents, only one attributed their unemployment to the COVID-19 pandemic. Additionally, among the 24 employed respondents, 19 were working full-time, while 5 were working part-time.

Table S1: Educational level and professional situation during COVID-19.

**Educational Level**	**High School or less**	**Bachelor's Degree**	**Master's Degree**	**Doctorate Degree**	**Other (Professional Course)**
***n* (Percentage %)**	11 (34.4%)	14 (43.8%)	4 (12.5%)	2 (6.3%)	1 (3.1%)

**Professional Situation**	**Secure job**	**Job at risk**	**Unemployed**
***n* (Percentage%)**	22 (68.8%)	2 (6.3%)	8 (25.0%)

Note: *N*=32

The sociodemographic questionnaire assessed if the respondents were suffering from mental health problems. Twelve respondents revealed having a psychiatric diagnosis, 9 of them had a diagnosis of depression, and 3 of anxiety. Nine of those were receiving psychotherapeutic and/or pharmacological treatment. Moreover, out of those 32 individuals, 5 reported feeling the need to contact mental health services during the COVID-19 pandemic.

Regarding COVID-19 situation we added a Table S2 summarizing additional information of current health status, prophylactic quarantine and time passed in home.

Table S2: COVID-19 context for respondents.

**COVID-19 Context**			
**Respondent Health Status**			
I was tested and the result was negative	I have been infected but I	I am asymptomatic and	I had symptoms, took the test, and it was negative.

	am no longer infected.	don't think I might be infected.	
9 (28.1%)	6 (18.8%)	16 (50%)	1 (3.1%)

**Prophylactic Quarantine**				
I voluntarily placed myself in prophylactic isolation	I am voluntarily in prophylactic isolation	I was in prophylactic isolation based on medical advice	I am in prophylactic isolation on medical advice	I have never undergone prophylactic isolation
1 (3.1%)	1 (3.1%)	9 (28.1%)	1 (3.1%)	20 (62.5%)

**At this moment…**			
I am always at home	I am mostly at home and only go out occasionally for personal or work situations	I mostly go out to work/classes	I do my normal life; I go out for work/classes and leisure
2 (6.3%)	10 (31.3%)	9 (28.1%)	11 (34.4%)

Note: N=32

The results obtained for resilience dimensions seem to point out that caregivers had a moderate level of self-perception (M = 4.64 ± .21), future perception (M = 4.45 ± .33), social skills (M = 4.35 ± .21), family cohesion (M = 5.40 ± .16), social resources (M = 5.04 ± .20) and structured style (M = 4.64 ± .19). The figure S1 shows the RSA results for all resilience dimensions.

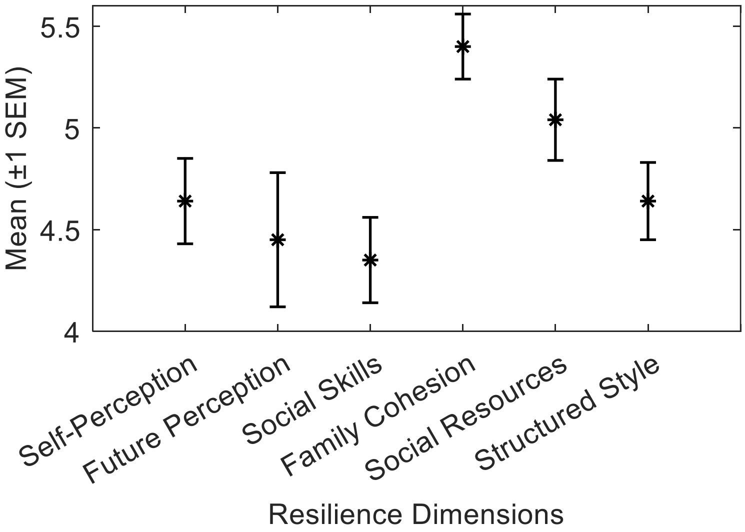

Figure S1: RSA results across resilience dimensions by *M* ± 1 *SEM*, with higher scores reflecting better resilience (*Min* = 1; *Max* = 7, per dimension) (*N* = 28).

The ABC results showed a range of scores in each subscale ranging between no behavioral problems to the existence of behavioral problems that are felt as severe. Figure S2 shows the results obtained in each scale of ABC.

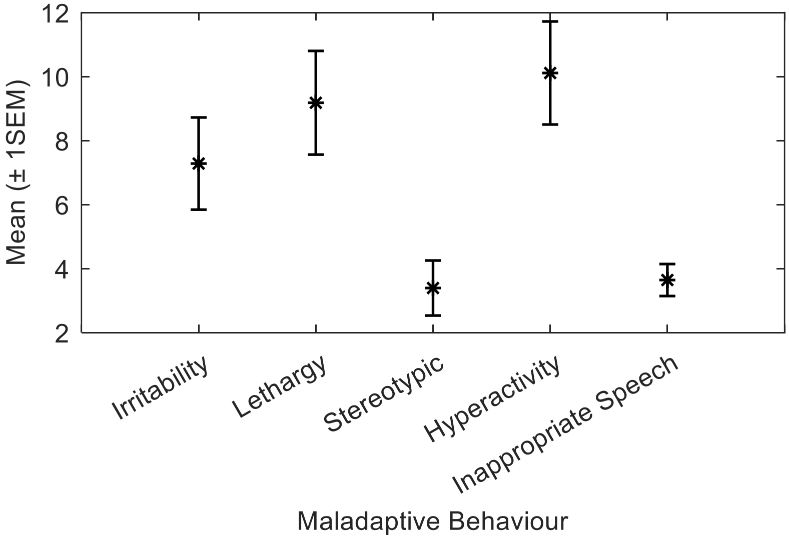

Figure S2: ABC characterization by maladaptive behavior subscales by *M* ± *SEM*, with higher scores indicating more severe behavioral problems (*N* = 28).

Materials

Sociodemographic Questionnaire

The sociodemographic questionnaire was composed by questions about the respondent. However, if the respondent identifies themselves as a caregiver of someone with a neurodevelopmental disorder, they were asked to fill out a subset of questionnaires presented in this study.

Individual characteristics:

1. Sex (options);

2. Age (open-ended response);

3. Nationality (open-ended response);

4. District where he/she lives (open-ended response);

5. If was living in Portugal Continental or in the Madeira or Azores Islas since 1 of March 2020? (yes or no);

Relational aspects:

1. Civil Status (options);

2. Relation Duration (open-ended response);

3. If have sons or daughters (yes or no) If yes,…

a. How many? (open-ended response);

b. Ages? (open-ended response);

c. Living with the respondent? (yes or no);

d. Indicate the ages of the sons/daughters living with you?;

e. Indicate the number of persons living together?;

Academic qualifications and work status:

1. Work situation (options);

2. Profession (open-ended response);

3. Academic qualifications (options);

Religious beliefs (options);

History of mental health problems:

1. At some point in your life, have you been diagnosed with a psychiatric disorder by a psychologist or psychiatrist? (Yes/no) If yes,…

a. What was the diagnosis? (options);

b. When was it made the diagnosis? (options);

c. Have you received psychological and/or psychiatric care (i.e., psychotherapeutic intervention)? (yes/no);

d. Have you received pharmacological therapy? (yes/no);

COVID-19 situation:

Since the National State of Emergency was declared on March 18, 2020...

1. Have you felt the need to contact mental health services (e.g., psychologists, psychiatrists, SNS24, etc.)? (yes or no) If yes…

a. Describe the type of contacts (open-ended response)

b. Evaluate the quality of their response (options)

2. Had to change residence? (yes or no) If yes…

a. Which was the reason to change residence. (options)

3. Health status regarding COVID-19 (options)

4. Quarantine situation (options)

5. In what situation are you presently? (options)

6. Are you familiar with professionals in the context of healthcare delivery? (yes or no) If yes…

a. Indicate the profession(s) of your family member(s). (options)

7. Are you a caregiver for a family member with Neurodevelopmental Disorders? (yes or no) If yes, …

a. Do you live with that person? (Yes or no)

b. What is your degree of relationship? (options)

c. What is the diagnosis of your family member? (options)

d. Indicate the age of this person (open-ended response)

Table S3: Overview of the measures applied to assess caregivers' psychopathology, resilience dimensions, burden and behavioral problems of the person with a neurodevelopmental disorder.

Self-report Measures	Main Goal(s)	Items Number (Response Time)	Response Scale	Minimum - Maximum Scores	Results Interpretation	Internal Consistency Portuguese Version [Original Version]
**Depression Anxiety Stress Scales 21 items (DASS-21)** (26,27)	Measure to assess the caregiver's psychopathology in three dimensions thinking about the past week:<br>1. Depression<br>2. Anxiety<br>3. Stress	21 Items<br>(Response time: 10 minutes)	4-point Likert scale:<br>0 (did not apply to me at all) to 3 (applied to me very much or most of the time).	0 - 21 (per scale)<br>0-63 (Total score)	The higher scores meaning higher level of a negative affective state.<br>Classification by scales:<br>Depression<br>Normal 0-4<br>Mild 5-6<br>Moderate 7-10<br>Severe 11-13<br>Extremely Severe 14 +<br>Anxiety<br>Normal 0-3<br>Mild 4-5<br>Moderate 6-7	1. Depression Cronbach Alpha = .85 [.91]<br>2. Anxiety Cronbach Alpha = .74 [.87]<br>3. Stress Cronbach Alpha =.81 [.91]

					Severe 8-9<br>Extremely Severe 10+<br><br>Stress<br>Normal 0-7<br>Mild 8-9<br>Moderate 10-12<br>Severe 13-16<br>Extremely Severe 17+	
**Resilience Scale for adults (RSA)**<br>(28-30)	Measure to evaluate different resilience dimensions thinking about the last month:<br><br>1. Self-perception (6 items)<br>2. Future planning (4 items)<br>3. Social competencies (6 items)<br>4. Structured style (4 items)<br>5. Family cohesion (6 items)<br>6. Social resources (7 items)	33 Items<br><br>(Response time: 10 minutes)	Semantic differential-type response format from 1 to 7	33-231 (Total)<br><br>1-7 (Per dimension)	The higher scores reflecting better resilience.	1. Self-perception Cronbach Alpha = .78<br>2. Future planning Cronbach Alpha = .75<br>3. Social competencies Cronbach Alpha = .72<br>4. Structured style Cronbach Alpha = .38<br>5. Family cohesion Cronbach Alpha = .81<br>6. Social resources Cronbach Alpha = .84<br>[Cronbach's alphas ranging from .76 to .87 for all factors]

**Revised Burden Measure (RBM)** (31,32)	Instrument to assess the burden in three dimensions:<br><br>1. Objective burden (6 items)<br>2. Subjective burden (5 items)<br>3. Relationship burden (5 items)<br>4. Uplifts related to the gratifications resulting from the informal care provision (6 items).	22 Items<br><br>(Response time: 3 minutes)	5-point Likert scale:<br><br>1 (Not at all) to 5 (A great deal)	6-30 (Objective Burden)<br><br>5-25 (Subjective burden)<br><br>5-25 (Relationship burden)<br><br>6-30 (Uplifts)<br><br>16 -80 (Total Burden)	The higher scores in the burden and/or uplifts indicate major alteration in that caregivers' life dimensions, as a consequence of the informal care provision.<br><br>Classifications:<br>Objective Burden<br>Low (6-10)<br>Average (11-18)<br>High (19-30)<br><br>Subjective Burden<br>Low (5-8)<br>Average (9-13)<br>High (14-25)<br><br>Relationship Burden<br>Low (5-5)<br>Average (6-10)<br>High (11-25)<br><br>Uplifts<br>Low (6-9)	1. Objective burden Cronbach Alpha = .87<br>2. Subjective burden Cronbach Alpha =.87<br>3. Relationship burden Cronbach Alpha = .83<br>4. Uplifts Cronbach Alpha = .81<br>5. Total Burden Cronbach Alpha = .92

					Average (10-16)<br>High (17-30)	
**Aberrant Behavior Checklist-Community (ABC-C)** (33,34,35,36)	Informant-based questionnaire to evaluate the maladaptive behavior in the last four weeks in five areas:<br>1. Irritability, agitation and crying (15 items)<br>2. Lethargy/social withdrawal (16 items)<br>3. Stereotypic (7 items)<br>4. Hyperactivity/ noncompliance (16 items)<br>5. Inappropriate speech (4 items)	58 Items<br>(Response Time 10-15 minutes)	4-point Likert scale:<br>0 (Not at all a problem) to 3 (The problem is severe in degree)	0-45 (Irritability, agitation and crying)<br>0-48(Lethargy/Social withdrawal)<br>0-21(Stereotypic)<br>0-48 (Hyperactivity/ noncompliance)<br>0-12 (Inappropriate speech)	Higher scores indicate more severe behavioral problems.	Brazilian-Portuguese Version translation used.<br>1. Irritability, agitation and crying [Cronbach Alpha = .91]<br>2. Lethargy/social withdrawal [Cronbach Alpha = .90]<br>3. Stereotypic [Cronbach Alpha = .84]<br>4. Hyperactivity/ noncompliance [Cronbach Alpha = .95]<br>5. Inappropriate speech [Cronbach Alpha = .77]

Exploratory Analysis Methods

Criteria for the selected measures and dimensions assessed in this study were based on findings about individual and family functioning dimensions that were affected during COVID-19 and that constitute central processes in family functioning. Specifically, this includes the family cohesion dimension, defined as cooperation, support, loyalty and stability, was considered because families during COVID-19 quarantine were strongly challenged to balance the togetherness and the individual space. Then, the social resources related to the social support that was discontinued during COVID-19, which have impacted on family and individuals' routines. Moreover, we also considered the caregiver structured style that is related to the capability of planning and managing time, which was crucial for caregivers that had to do their job from home and adjusted to the informal care provision that was required during pandemic times. In addition, we choose to include the caregiver's self-perception defined as the self-judgements about abilities, feeling of self-efficacy and the expectations adjustments to reality. In this line, we selected the relationship burden, because results from an exclusive relationship between the caregiver and the person in care can originates a negative psychological state. Additionally, we added subjective burden which is a measure of an overall negative affect resulting from the informal care provision. Finally, we introduced one behavior dimension from the person in care – irritability defined as tantrums, verbal outbursts, self-harm, and negative affect (1). Irritability has been pointed out as being highly associated with several dimensions of caregiver's burden, which was supported by previous studies suggesting that aggressive and disruptive behaviors are significant for family outcomes (2).

Two hierarchical multiple regressions were performed to identify protective and risk factors for the caregivers' wellbeing. The first hierarchical multiple regression was performed to explore which variables added will inform the prediction of the caregiver's depressive state obtained by DASS-21 depression scores. Therefore, the depression score was introduced as the outcome. The caregivers' age, sex, and existence of psychiatric diagnosis were introduced in the first block. Then, we introduced the following predictors in each following blocks family cohesion (RSA), social resources (RSA), structured style (RSA), relationship burden (RBM), subjective burden (RBM), and the irritability, agitation and crying score (ABC) of the individual with a neurodevelopmental disorder. There was linearity as assessed by partial regression plots and a plot of studentized residuals against the predicted values. Residuals were independent, as assessed by a Durbin-Watson statistic of 2.405. There was homoscedasticity, as assessed by visual inspection of a plot of studentized residuals versus unstandardized predicted values. There was no evidence of multicollinearity, as assessed by tolerance values greater than .1. There were no studentized deleted residuals greater than ±3 standard deviations, 5 leverage values greater than .5, and no values for Cook's distance above 1. The assumption of normality was met, as assessed by Q-Q Plot.

A second hierarchical multiple regression was done to determine if the addition of predictors self-perception (RSA), family cohesion (RSA), and caregiver's structure style (RSA) during the COVID-19 pandemic could improve the prediction of caregiver's overall burden (outcome) and above age, sex, and previous psychiatric diagnosis alone. Linearity was assessed by partial regression plots and a plot of studentized residuals against the predicted values. There was independence of residuals obtained by the Durbin-Watson statistic of 1.293. There was homoscedasticity, as evaluated by visual inspection of a plot of studentized residuals versus unstandardized predicted values. There was no evidence of multicollinearity, as verified by tolerance values greater than .1. There were no studentized deleted

residuals greater than ±3 standard deviations, two leverage values greater than .5, and no values for Cook's distance above 1. The assumption of normality was met, as assessed by Q-Q Plot.

Exploratory Analysis Results

Caregiver's Depressive State Prediction

We developed 7 alternative models. In the first model the predictors were the caregivers' age, sex and the existence of a previous psychiatric disorder diagnosis. This model was not statistically significant, $F(3, 23) = .888$, $p = .462$. In the second model, was added to the previous model the predictor family cohesion. This second model was statistically significant, $F(4, 22) = 4.183$, $p < .05$. We found that the family cohesion addition to model 2 led to a statistically significant increase in R^2 of .328, $F(1, 22) = 12.710$, $p<.05$. Therefore, 32.8% of the variance explained in the DASS-21 depression scores can be attributed to the family cohesion scores at RSA. This result indicates that family cohesion was significantly associated with the caregiver's depressive sate ($B = -4.147$, $t = -3.565$, $p = .002$). Therefore, the change in one unit for family cohesion score in RSA will result in - 4.147 (95% CI, -6.560 to -1.735) in the value of DASS-21 depression scores. The third model included the previous predictors and in addition the social resources, which was statistically significant, $F(5, 21) = 3.936$, $p < .05$. However, the addition of this predictor was not statistically significant. The model 4 and 5 included the caregiver's structured style and the relationship burden, respectively. These two models showed to be statistically significant (Model 4, $F(6, 20) = 3.355$, $p < .05$; Model 5, $F(7, 19) = 3.271$, $p < .05$), but not the predictors added. Furthermore, the model 6 showed to be statistically significant, $F(8, 18) = 4.713$, $p < .05$. The addition of the subjective burden to the model 6 resulted in a statistically significant increase in R^2 of .130, $F(1, 18)= 7.26$, $p <.05$. Consequently, 13% of the variance in the DASS-21 depression scores will be explained by the subjective burden assessed by the RBM. This result means that by the addition of one unit in subjective burden score assessed by the RBM, there will be a change of .485 (95% CI, .107 to .863) in DASS-21 depression scores. This significant association ($B = .485$, $t = 2.694$, $p < .05$) reflects a positive relationship between the subjective burden and depression (i.e., higher scores in subjective burden will predict higher scores in depression). Finally, the full model (model 7) to predict the caregiver's depressive state was statistically significant, $R^2 = .679$, $F(9, 17) = 3.987$, $p<.05$; *adjusted* $R^2 = .508$. Table S4 details each regression model obtained.

Table S4. Hierarchical Multiple Regression results for caregiver's depressive state.

	Depression													
	**Model 1**		**Model 2**		**Model 3**		**Model 4**		**Model 5**		**Model 6**		**Model 7**	
**Variable**	***B***	**β**	***B***	**β**	***B***	**β**	***B***	**β**	***B***	**β**	***B***	**β**	***B***	**β**
**Constant**	-11.238		18.684		19.149		23.071		26.729		15.882		15.857	
**Age**	.101	.151	.004	.006	.032	.049	-.014	-.021	-.017	-.026	-.002	-.003	.008	.012
**Sex**	5.201	.256	1.598	.079	1.244	.061	.939	.046	1.838	.090	-.364	-.018	-.160	-.008
**PD**	1.091	.099	4.041	.366	3.299	.299	2.657	.241	2.685	.243	1.540	.140	1.223	.111
**FC**			-4.147	-.658	-2.546	-.404	-1.286	-.204	-1.894	-.300	1.350	.214	1.502	.238
**SR**					-1.724	-.329	-2.016	-.385	-1.722	-.328	-2.619	-.500	-2.654	-.506
**SS**							-1.193	-.230	-1.401	-.270	-1.946	-.375	-2.096	-.403
**RB**									-.293	-.240	-.472	-.387	-.509	-.417
**SB**											.485	.560	.459	.530
**I**													.048	.069
R^2	.104		.432		.484		.502		.547		.677		.679	
F	.888		4.183*		3.936*		3.355*		3.271*		4.713*		3.987*	
ΔR^2	.104		.328		.052		.018		.045		.130		.002	
ΔF	.888		12.710*		2.108		.717		1.881		7.260*		.089	

Note: $N = 27$, $*p < .05$, $**p < .001$. PD: Psychiatric Diagnosis; FC: Family Cohesion (RSA); SR: Social Resources (RSA); SS: Structured Style (RSA); RB: Relationship Burden (RMB); SB: Subjective Burden (RMB); I: Irritability (ABC)

Caregiver's Overall Burden Prediction

We analyzed four alternative models by introducing in the first model age, sex, and the presence of a previous psychiatric diagnosis. This model was not statistically significant, $F(3, 23) = 1.964$, $p = .148$. In the second model, we added the caregiver's self-perception as a predictor of caregiver's overall burden. We found that this model was statistically significant ($F(4, 22) = 2.860$, $p < .05$) and that predictor addition led to a statistically significant increase in R^2 of .138, $F(1, 22) = 4.620$, $p < .05$. This means that caregiver's self-perception explains 13.8% of the variance in the caregiver's overall burden. This significantly negative association between caregiver's self-perception and overall burden ($B = -5.884$, $t = -2.149$, p < .05) reflects that the increasing in the self-perception scores lead to lower overall burden. In addition, the change in one unit of caregiver's self-perception score will result in a change of -5.884 (95% CI, -11.560 to -.207) in overall burden score. In the third model was added to the previous predictors the family cohesion. This model was statistically significant, $F(5, 21) = 4.314$, $p < .05$. Furthermore, the addition of family cohesion to the prediction of the caregiver's also led to a statistically significant increase in R^2 of .165, $F(1, 21) = 7.007$, $p < .05$. Therefore, 16.5% of the variance found in the caregiver's overall burden is explained by the family cohesion. Moreover, this significant negative relationship ($B = -10.740$, $t = -2.647$, $p < .05$) between the family cohesion and the caregiver's overall burden means that the higher the family cohesion there is less caregiver's overall feeling of burden. Additionally, the family cohesion score change in one unit will result in a change of -10.740 (95% CI, -19.177 to -2.302) in the caregiver's overall burden score. The full model of sex, age, psychiatric diagnosis, self-perception, family cohesion, and caregiver's structured style (Model 4) was statistically significant, $R^2 = .507$, $F(6, 20) = 3.426$, $p < .05$; *adjusted* $R^2 = .359$. Table S5 details each regression model.

Table S5: Hierarchical Multiple Regression results for caregiver's overall burden.

Caregiver's Overall Burden								
	**Model 1**		**Model 2**		**Model 3**		**Model 4**	
**Variable**	***B***	**β**	***B***	**β**	***B***	**β**	***B***	**β**
**Constant**	2.381		52.585		92.631		93.460	
**Age**	.061	.030	-.136	-.067	-.239	-.119	-.252	-.125
**Sex**	24.815	.404	18.549	.302	13.892	.226	13.856	.226
**PD**	-10.199	-.306	-11.155	-.335	-2.801	-.084	-2.885	-.087
**SP**			-5.884	-.399	-1.496	-.101	-1.413	-.096
**FC**					-10.740	-.564	-10.529	-.553
**SS**							-.333	-.021
R^2	.204		.342		.507		.507	
F	1.964		2.860*		4.314*		3.426*	
ΔR^2	.204		.138		.165		.000	
ΔF	1.964		4.620*		7.007*		.006	

Note: *N* = 27, **p* < .05, ***p* < .001. PD: Psychiatric Diagnosis; SP: Self-Perception (RSA); FC: Family Cohesion (RSA); SS: Structured Style (RSA); Caregiver's Burden is a measure of all burden measures sum (objective, subjective and relationship burden).

Hierarchical Multiple Regression Assumptions

A third hierarchical multiple regression was done with the previous added significant predictors: family cohesion and subjective burden. Linearity was assessed by partial regression plots and a plot of studentized residuals against the predicted values. There was independence of residuals obtained by the Durbin-Watson statistic of 2.402. There was homoscedasticity, as evaluated by visual inspection of a plot of studentized residuals versus unstandardized predicted values. There was no evidence of multicollinearity, as verified by tolerance values greater than .1. There was one studentized deleted residuals greater than ±3 standard deviations, no leverage values greater than .5, and no values for Cook's distance above 1. The assumption of normality was met, as assessed by Q-Q Plot.

A fourth hierarchical multiple regression was performed including the previous added significant predictors: self-perception and family cohesion. Linearity was assessed by partial regression plots and a plot of studentized residuals against the predicted values. There was independence of residuals obtained by the Durbin-Watson statistic of 1.232. There was homoscedasticity, as evaluated by visual inspection of a plot of studentized residuals versus unstandardized predicted values. There was no evidence of multicollinearity, as verified by tolerance values greater than .1. There were any studentized deleted residuals greater than ±3 standard deviations, no leverage values greater than .5, and no values for Cook's distance above 1. The assumption of normality was met, as assessed by Q-Q Plot.
